# Low-cost microvascular phantom for photoacoustic imaging using loofah

**DOI:** 10.1117/1.JBO.30.1.016006

**Published:** 2025-01-20

**Authors:** Jinhua Xu, Yixiao Lin, Sanskar Thakur, Haolin Nie, Lukai Wang, Quing Zhu

**Affiliations:** a Washington University in St. Louis, Department of Biomedical Engineering, St. Louis, Missouri, United States; b Washington University in St. Louis, Imaging Science Program, St. Louis, Missouri, United States

**Keywords:** photoacoustic imaging, microvascular phantom, tumor phantom loofah material, acoustic-resolution photoacoustic microscopy, optical-resolution photoacoustic microscopy

## Abstract

**Significance:**

Existing photoacoustic phantoms are unable to mimic complex microvascular structures with varying sizes and distributions. A suitable material with structures that mimic intricate microvascular networks is needed.

**Aim:**

Our aim is to introduce loofah as a natural phantom material with complex fiber networks ranging from 50 to 300  μm, enabling the fabrication of phantoms with controlled optical properties comparable to those of human microvasculature.

**Approach:**

By introducing a controllable chromophore into the loofah material, we controlled its absorption properties. The loofah’s vasculature-mimetic capabilities and stability in photoacoustic signal generation were evaluated using co-registered ultrasound, acoustic-resolution photoacoustic microscopy (ARPAM), and optical-resolution photoacoustic microscopy (ORPAM).

**Results:**

ORPAM results confirmed the loofah’s ability to control chromophore distribution, leading to consistent and regulated photoacoustic signals. ARPAM results demonstrated that the loofah phantom effectively replicates vascular structures, exhibiting superior performance in mimicking microvascular networks compared with commonly used tissue-mimetic phantoms. The dominant diameter range of the phantom’s microvasculature was between 100 and 250  μm, aligning well with the targeted range and facilitating meaningful comparisons with human vascular structures.

**Conclusions:**

The loofah material provides a low-cost and effective method for creating submillimeter microvascular phantoms for photoacoustic imaging. Its exceptional morphology and customizability allow it to be shaped into various vascular network configurations, enhancing the fidelity of phantom imaging and assisting in system calibration and validation. In addition, data obtained from this realistic microvascular phantom can offer greater opportunities for training machine learning models.

## 1 Introduction

Tissue-mimetic phantoms, capable of emulating the desired soft tissue properties, provide a clinically relevant measurement and imaging environment for the development and validation of medical imaging systems. An emerging class of preclinical photoacoustic (PA) imaging systems that have shown promise in cancer diagnosis and monitoring^1^^,^^2^ rely on the microvascular patterns observed through PA imaging to differentiate healthy from diseased tissue. Thus, the ability to robustly construct a tissue-mimetic phantom with precisely controlled microvascular distribution is valuable for the advancement of PA imaging systems.

Traditional PA phantoms made from blood tubings^3^ lack the fine microvasculature pattern, limiting their effective simulation of the *in vivo* imaging conditions and thus their ability to validate and characterize preclinical PA imaging systems, especially high-resolution photoacoustic microscopy systems. Advancements in fabrication methods have attempted to address these limitations by incorporating finer features and more complex geometries into phantoms. Techniques ranging from simple tubing or channel embedding^3^^,^^4^ to advanced methods such as 3D printing^5^ and cutting-edge approaches such as hydrogel printing^6^ have been explored to create vascular structures. However, channel embedding techniques are limited to simple patterns,^3^ and 3D-printed small-region vasculature presents challenges, particularly in post-printing cleaning of material supports used by PolyJet printers, especially for tortuous phantoms or vessels smaller than 2 mm.^5^ Even low-cost bioprinters typically cost thousands of dollars, with consumables adding significantly to the expense.^7^ Overall, these methods face challenges related to resolution limits, material properties, and replicating the optical and acoustic characteristics essential for accurate photoacoustic imaging.

In this study, we present a new approach for fabricating low-cost vascular phantoms that closely mimic the morphology and complexity of submillimeter microvascular networks. By utilizing loofah materials and precision fabrication techniques, we have developed phantoms with vessel diameters in the range of 50 to 300  μm and intricate branching patterns. These phantoms are designed to replicate the microvasculature as well as the optical and acoustic properties of biological tissues. To the best of our knowledge, this is the first time that loofah materials have been utilized for photoacoustic imaging phantoms. We believe loofah phantoms provide an excellent low-cost tool for bio-optics researchers to characterize preclinical vascular imaging systems.

## 2 Materials and Methods

### 2.1 Vascular-Mimetic Material Description

Loofah sponge is the fibrous vasculature, or xylem, of a loofah plant, originally used for distributing water and nutrients inside the plant via capillary action. Similar to leaf phantoms, which are commonly used for characterizing microscopic imaging systems, the vessels inside a loofah sponge have a range of diameters, shapes, and directions. However, unlike leaf phantoms, loofah sponges inherently provide a volumetric scaffold, making it more amenable to 3D imaging. An appealing property of loofah, crucial to the subsequent phantom construction, is that once dried, a loofah sponge can rapidly absorb any solution of interest and spread it uniformly throughout its vessels.

**Fig. 1:**
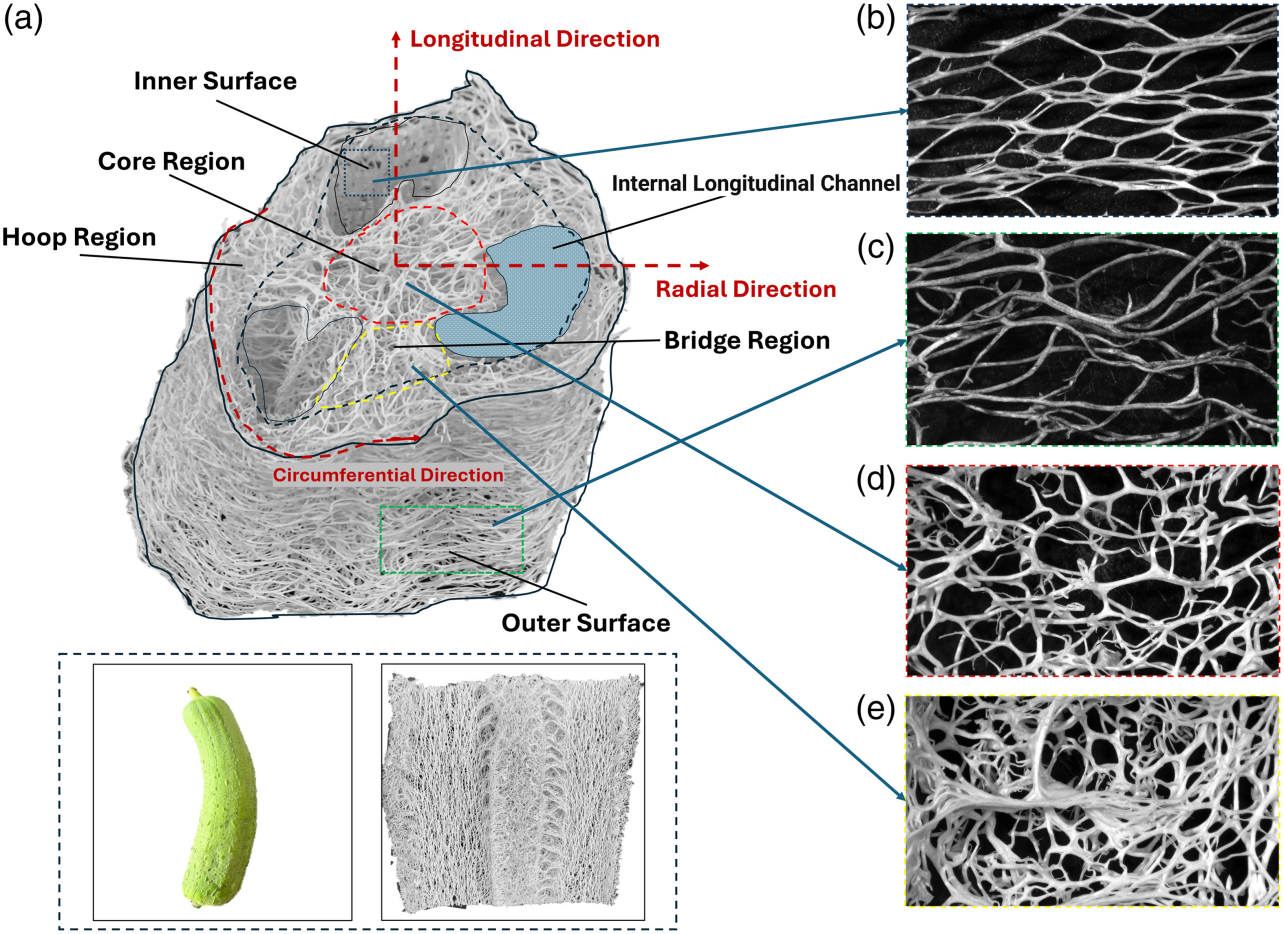
Cross-sectional view of loofah with expanded details of specific regions. (a) Defined structural regions of the loofah. (b) Inner surface on the hoop region side. (c) Outer surface of the hoop region. (d) Cross-sectional view of the core region. (e) Bridge region connecting the core and hoop regions. Inset images: (left) *Luffa* gourd and (right) vertical cross-section illustrating the overall loofah structure.

The loofah sponge contains three distinct regions: the hoop, the bridge, and the core, as illustrated in Fig. 1. Further, the inner and outer sides of the hoop are differentiated because the vessels on these opposite sides are orthogonally oriented. The fibers forming the outer surface of the loop region grow in the circumferential direction. Short longitudinal fibers interconnect the long circumferential fibers to support the 3D structure. In contrast, the inner surface of the loop region is mainly composed of longitudinal fibers, interconnected by shorter circumferential fibers. The cylindrical core at the center of loofah is composed of disordered fibers [see Fig. 1(d)]. The bridge part and the inner surfaces on the core side are longitudinally divided into elliptical chambers formed by irregular flat fibers distinct from the peripheral fibers, as shown in Fig. 1(e). This diversity of structures and architectures enables a variety of applications. In this study, the outer surface of the hoop region was utilized to mimic a flat vascular distribution, whereas the core region was employed to mimic radial vascular distributions.

### 2.2 Tissue-Mimetic Material Description

The loofah vasculature was embedded in hydrogels, such as gelatin and agar, due to their low inherent optical and acoustic attenuation. To match the optical and acoustic scattering properties of biological tissue, ${\mathrm{TiO}}_{2}$ particles and glass microspheres with a diameter range between 25 and 100  μm were added to the agar solution, respectively.

### 2.3 Selection of the Chromophore Solution

The chromophore solutions should satisfy two requirements. First, it must be water-based to support capillary action. Second, it must be waterproof once it is dry to prevent the chromophore from entering and dissipating in hydrogels through osmosis. In this study, waterproof India ink was used as the primary contrast agent.

### 2.4 Morphological Study of Loofah Vasculature

Significant morphological differences exist among the various regions of loofah. To characterize and quantify the vasculature of the different regions to enable the selection of the most appropriate region for different types of phantoms, we imaged each region with a stereo microscope (S9D, Leica, Wetzlar, Germany) and used ImageJ to quantify four parameters: number density of branch points, total vessel length per unit area, average vessel diameter, and vessel density.^8^^,^^9^ The quantification results were obtained from three different loofahs of similar size, sourced from three separate batches.

### 2.5 Phantom Fabrication Protocol

**Fig. 2:**
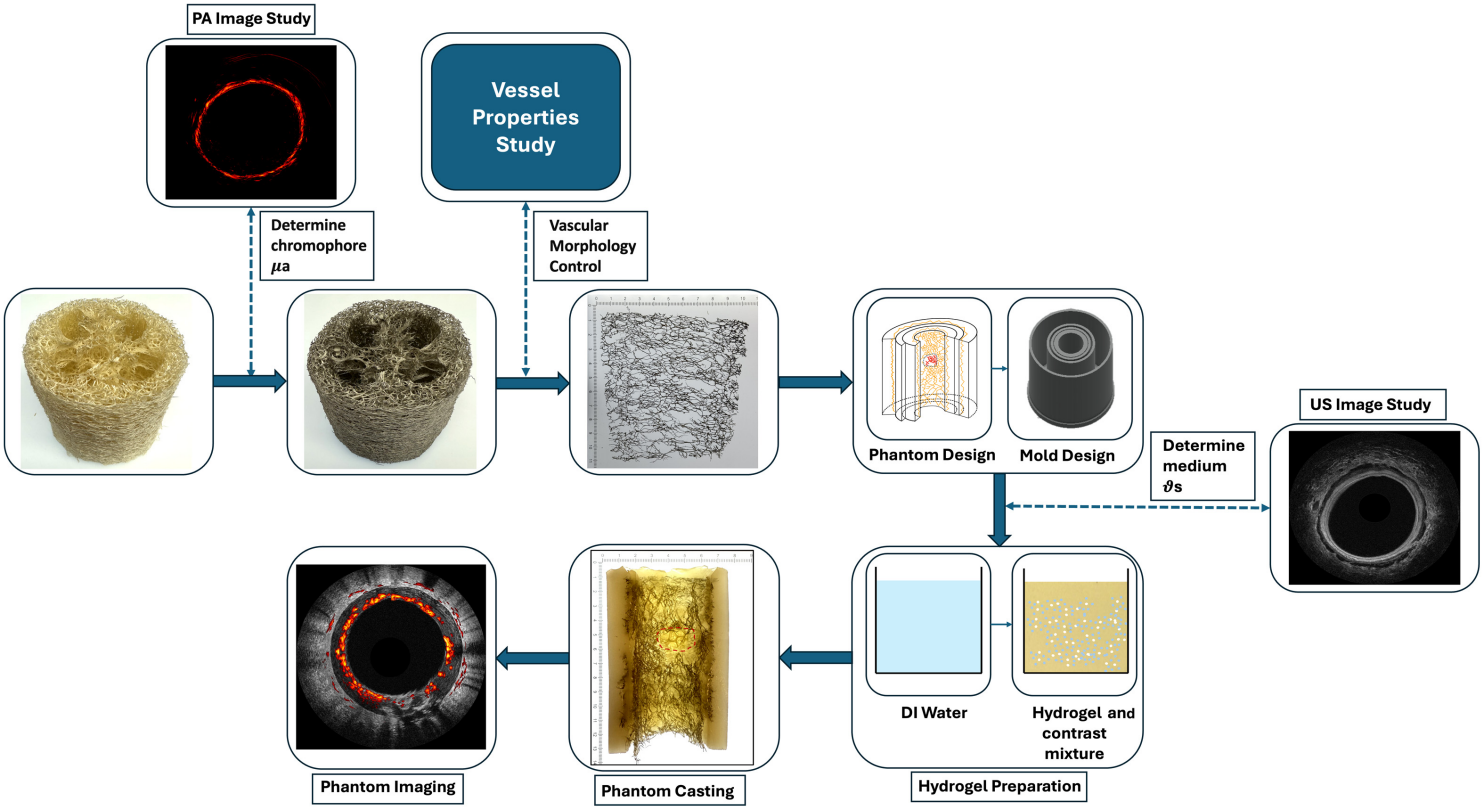
Flow chart of phantom fabrication protocol. μa: light absorption coefficient; ϑs: acoustic scattering coefficient.

Because loofah provides a range of shapes, sizes, and structures, it could be designed to mimic the microvasculature of different human organs and diseased tissue. Figure 2 illustrates the general protocol for loofah phantom fabrication. This protocol includes five steps: (1) select and study the target organ, (2) prepare fiber materials based on the photoacoustic properties of the target organ, (3) trim and shape the loofah material according to the blood vessel morphology of the target organ, (4) prepare a medium with similar optical and acoustic properties as the target organ tissue, and (5) perform phantom casting. The following sections describe the detailed design procedures of two loofah phantoms for two specific applications: Sec. 2.5.1 describes a planar phantom suitable for benchtop microscopic studies, and Sec. 2.5.2 describes a tubular phantom applicable to endoscopic imaging in clinical settings.

#### 2.5.1 Design of planar microscopic phantom

The design of a planar microscopic phantom involves casting loofah fibers onto the surface of clear agar. The loofah fibers were cut from the hoop’s outer surface because they represented a dense distribution of microvessels. The phantom was imaged with an in-house optical-resolution photoacoustic microscope, described in a previous publication.^10^

#### 2.5.2 Design of endoscopic photoacoustic phantom

**Fig. 3:**
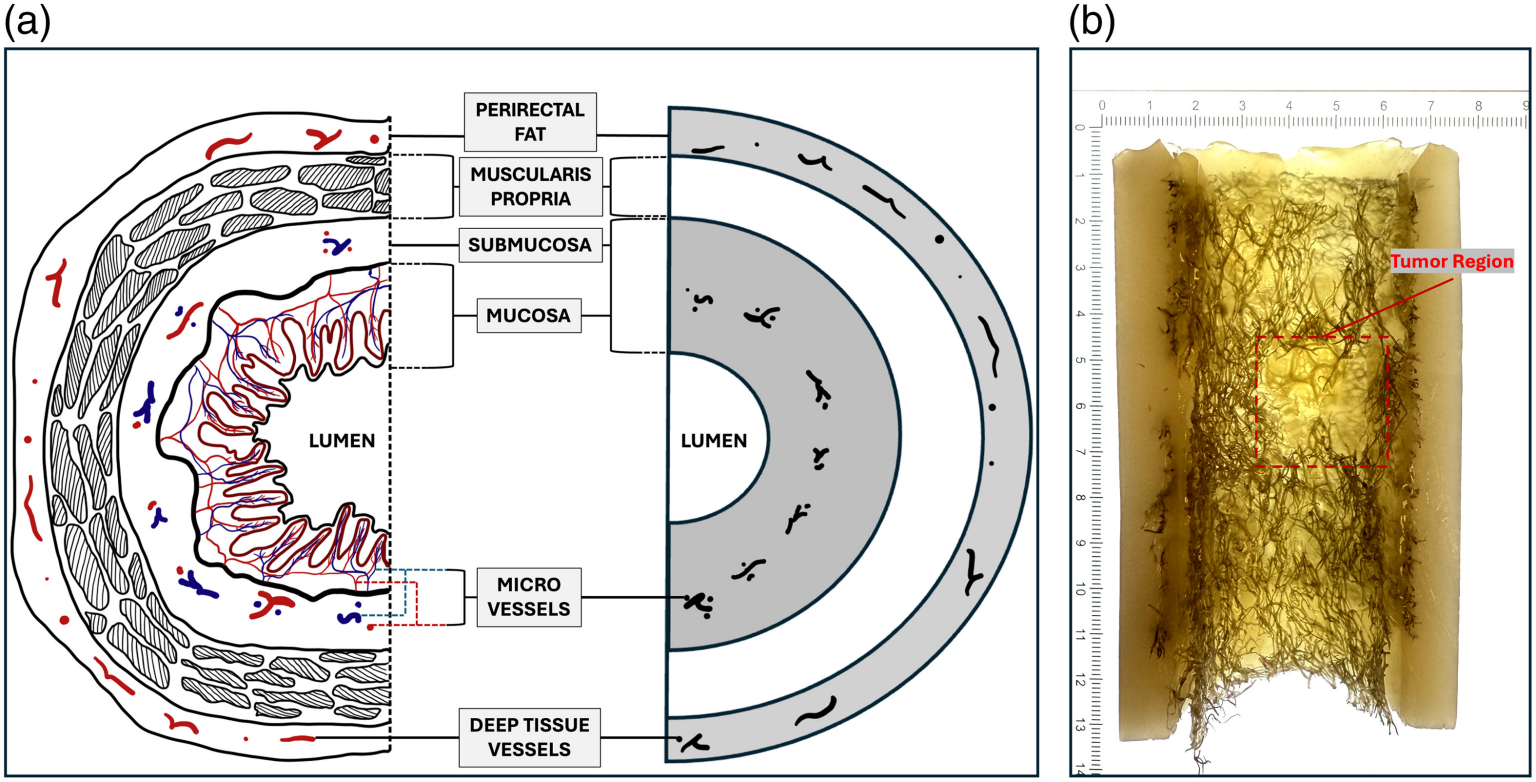
ARPAM phantom design demonstration. (a) Schematic representation of the cross-section, illustrating the three-layer structure of the phantom in comparison with the cross-sectional anatomy of the human rectum. (b) Vertical cross-section of the fabricated phantom model.

As illustrated in Fig. 3, the endoscopic phantom was cast into a tubular shape with three distinct layers corresponding to the three intestinal tissue layers. The first layer closest to the lumen mimics the mucosal and submucosal tissues that are rich in micro- and capillary blood vessels. The loofah fibers in this layer come from the outer surface of the hoop region, with their primary fiber orientation parallel to the central axis of the cylindrical phantom. The second layer consists solely of hydrogel and serves as the muscularis propria. The third layer mimicked perirectal fat and nearby connective tissue contains larger blood vessels supplying the intestinal system. Therefore, in this layer, the loofah fibers are again from the outer surface of the hoop region, but with their primary fiber orientation perpendicular to the central axis of the cylinder. To mimic an invasive adenocarcinoma, a tumor phantom made from the core region of the loofah was grafted into the layered phantom to mimic malignant invasion through the muscularis propria. This tumor phantom was fabricated with a lower dose of chromophore, because lower photoacoustic signal and lower microvascular density have been observed in malignant rectal lesions compared with healthy tissue, due to the rapid growth of cancerous cells outpacing the rate of angiogenesis, leading to pockets of low oxygenation and nutrient supply inside the tumor and subsequent necrosis.^11^ Table 1 summarizes the composition and structural properties of different layers. The phantom was imaged with an in-house acoustic-resolution photoacoustic endoscopic probe, described in a previous publication.^12^

**Table 1:** Composition and structural properties of layered medium.

Layers	Thickness (mm)	Medium type	${\mathrm{TiO}}_{2}$ (mg/ml)	Glass bead (mg/ml)	Contain loofah vasculature (yes/no)
Layer 1	2	10 wt% gelatine	0.5	4	Yes
Layer 2	2	10 wt% gelatine	0.25	1	No
Layer 3	10	3.5 wt% agar	0	40	Yes

## 3 Results

### 3.1 Quantitative Analysis of Loofah Morphology

**Fig. 4:**
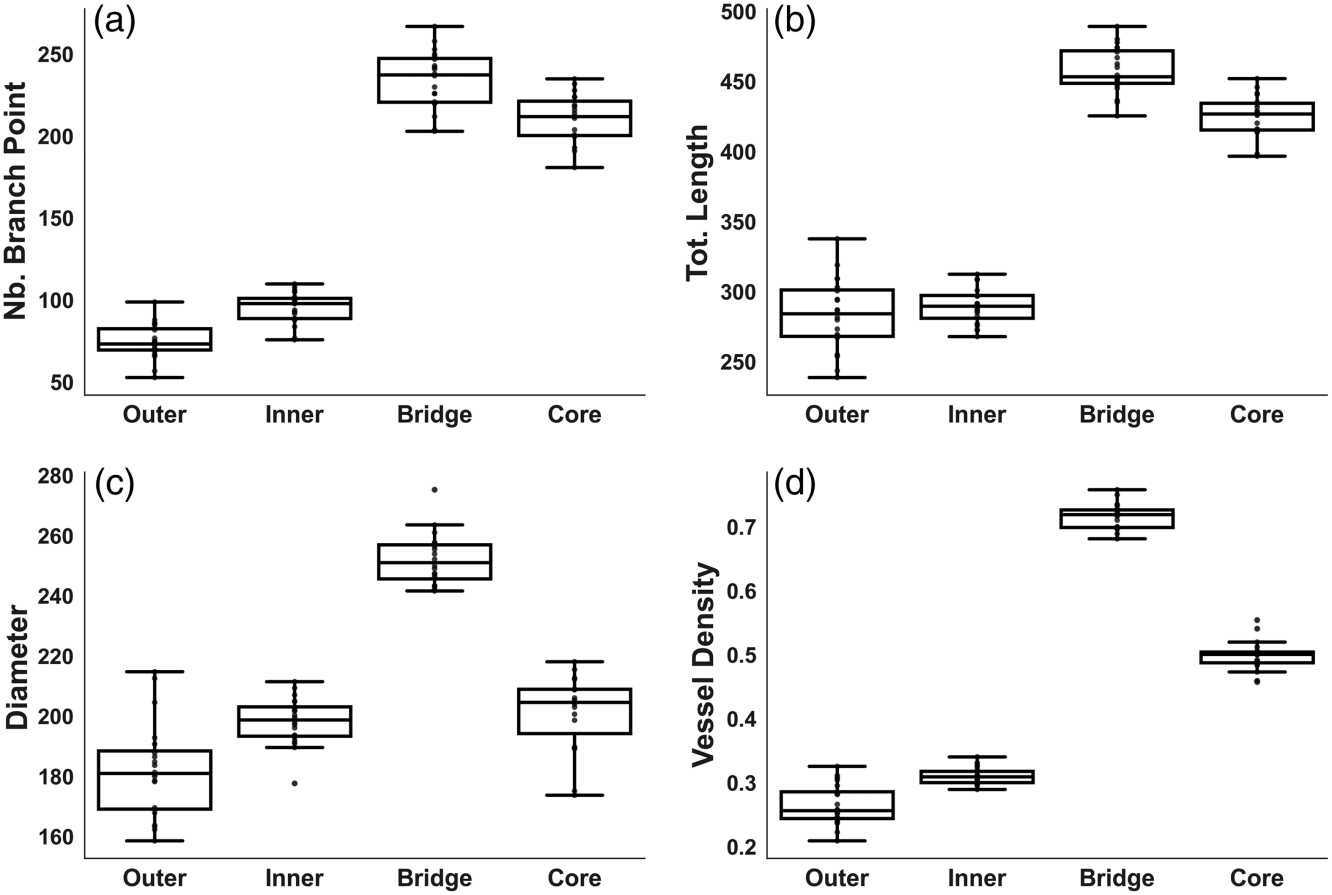
Boxplots of important markers used to show morphological differences. (a) Number of master junctions. (b) Total length. (c) Diameter. (d) Vessel density.

Table 2 presents the mean values of various quantitative morphological markers from different regions of loofah. Figure 4 illustrates the distributions of these morphological markers, comparing fibers from different regions of the loofah skeleton. Although both the inner and outer surfaces exhibit flat-distributed fiber vasculature in microscope images of the same size, the outer surface has fewer number of branch points (74 versus 95) compared with the inner surface, but a similar total vessel length (283.7 mm versus 289.6 mm). This indicates that the fiber length between branch points is greater for the outer surface. Consequently, the outer surface offers higher morphological customizability by allowing control over branch point removal. In addition, being composed of the thinnest fiber vasculature, the outer surface can be easily shaped to mimic submillimeter flat-distributed human microvasculature.

**Table 2:** Mean morphological parameters from different parts of the loofah skeleton.

Morphological markers	Outer surface	Inner surface	Bridge	Core
Number density of branch points	74	95	234	211
Total length of vessels (mm)	283.7	289.6	457.8	425.5
Vessel diameter (μm)	182.2	198.3	252.3	201.4
Vessel density (%)	26.5	31.1	71.6	49.9

The core and bridge regions are both dense fibrous areas. Despite having a similar number of branch junctions (234 versus 211) and total vessel lengths (457.8 mm versus 425.5 mm), their high vessel densities originate from distinctly different morphologies. The comparable number densities of branch points and total vessel lengths imply that the segment lengths between branch points are similar. In the core region, fiber segments and branch points are distinctly spaced apart. In contrast, the bridge region’s exceptionally high average vessel density (71.6%) and diameter (252.3  μm) result from its unique fiber bundles, which resemble groups of blood vessels—including arteries, veins, and capillaries—found in the human body.

#### 3.1.1 Distribution of vessel diameter

**Fig. 5:**
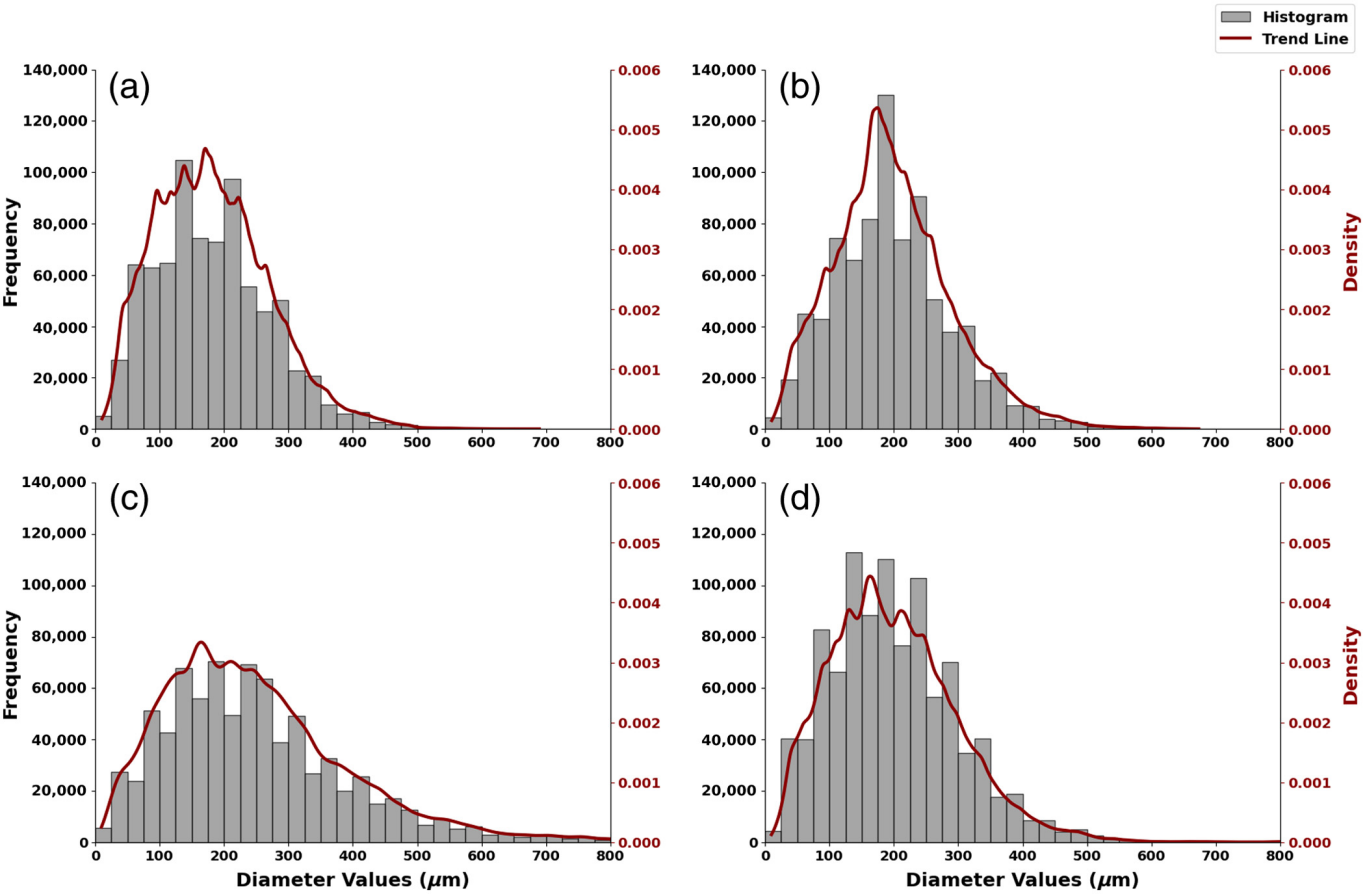
Histograms and trendlines of four major loofah regions. (a) Outer surface. (b) Inner surface. (c) Bridge region. (d) Core region.

In the statistical analysis of fiber diameters across four regions, prominent fluctuations are observed on the outer surface of the hoop region, with multiple small peaks in the 100 to 200  μm range [Fig. 5(a)]. This multi-peak phenomenon suggests the presence of subpopulations within the vascular network, underscoring the complexity of this region. In contrast, the inner region displays a steep decline following its peak [Fig. 5(b)], indicating that most diameters are concentrated around the peak with fewer larger outliers. This suggests less variation, as values are clustered in a narrower range, consistent with observations in Figs. 1(b) and 1(c).

The bridge region demonstrates a more complex vascular network, characterized by a broad distribution in the 100- to 300-μm range [Fig. 5(c)]. The larger standard deviation and extended tail indicate a wide range of vessel types and sizes. The slower decline after the peak, along with the extended tail of larger diameters, suggests a broad distribution of vessel diameters compared with the other regions. With diameters ranging from 9.79 to 1360.83  μm (Table 3), the sample includes both small microvessels and larger vascular bundles, as shown in Fig. 1(e), where different vessel types intertwine to form thicker, bundle-like structures.

**Table 3:** Summary of statistical metrics for regional diameter measurements.

Region	Mean (μm)	SD (μm)	Mode (μm)	Median (μm)	Min Diam. (μm)	Max Diam. (μm)
Outer surface	181.19	87.86	169.7	169.7	10.61	689.39
Inner surface	198.07	90.58	165.75	186.47	10.36	673.35
Bridge	252.14	151.41	156.64	225.17	9.79	1360.83
Core	199.47	97.86	158.38	188.08	9.9	999.79

In the core region, a multimodal distribution in the 100- to 250-μm range [Fig. 5(d)] reflects a network predominantly composed of smaller vessels. The relatively lower standard deviation and mean suggest that the vascular network within this region is more homogeneous, primarily consisting of smaller vessels. However, larger vessels are present, as indicated by the extended tail, though they occur less frequently, aligning with observations in Fig. 1(d).

### 3.2 Optical Resolution Photoacoustic Microscopy (ORPAM) Imaging

**Fig. 6:**
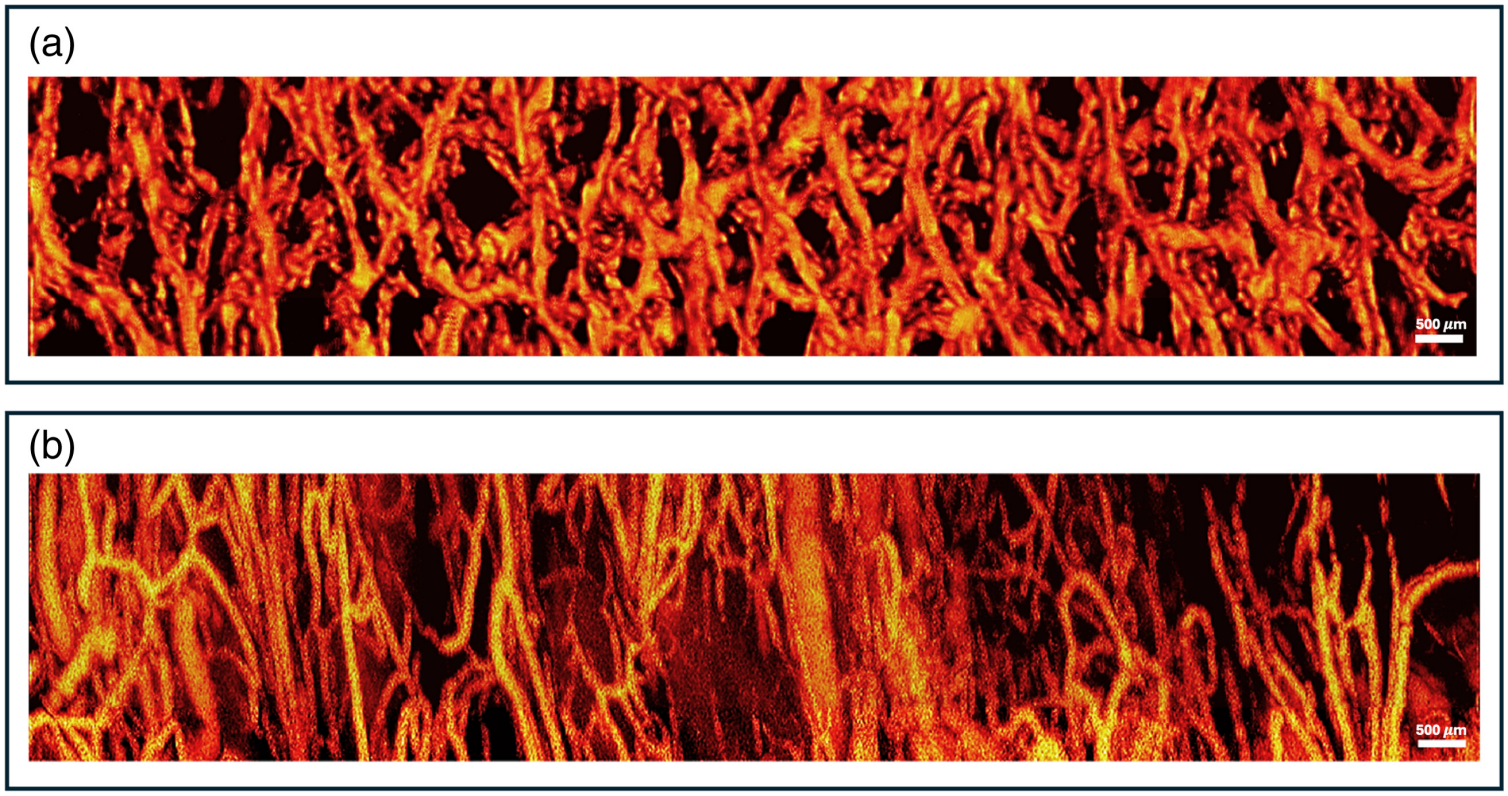
Example ORPAM image of (a) loofah phantom and (b) *ex vivo* ovarian specimen. Scale bar: 500  μm.

Figure 6(a) shows an example image of loofah fibers over a 3  mm×17  mm region, imaged with ORPAM. Uniform patterns were seen throughout the entire vascular network of the loofah phantom. Loofah’s microvascular patterns were then compared with those from a healthy human ovarian specimen, as shown in Fig. 6(b). The imaged region was chosen to be the connective tissue next to the uterus that supplies blood to the ovaries and uterine tubes and was therefore rich in blood vessels. It was observed that, even on a microscopic scale, loofah fibers successfully mimicked the density and directionality of tissue microvasculature.

### 3.3 Endoscopic Phantom Imaging

#### 3.3.1 B-mode imaging

**Fig. 7:**
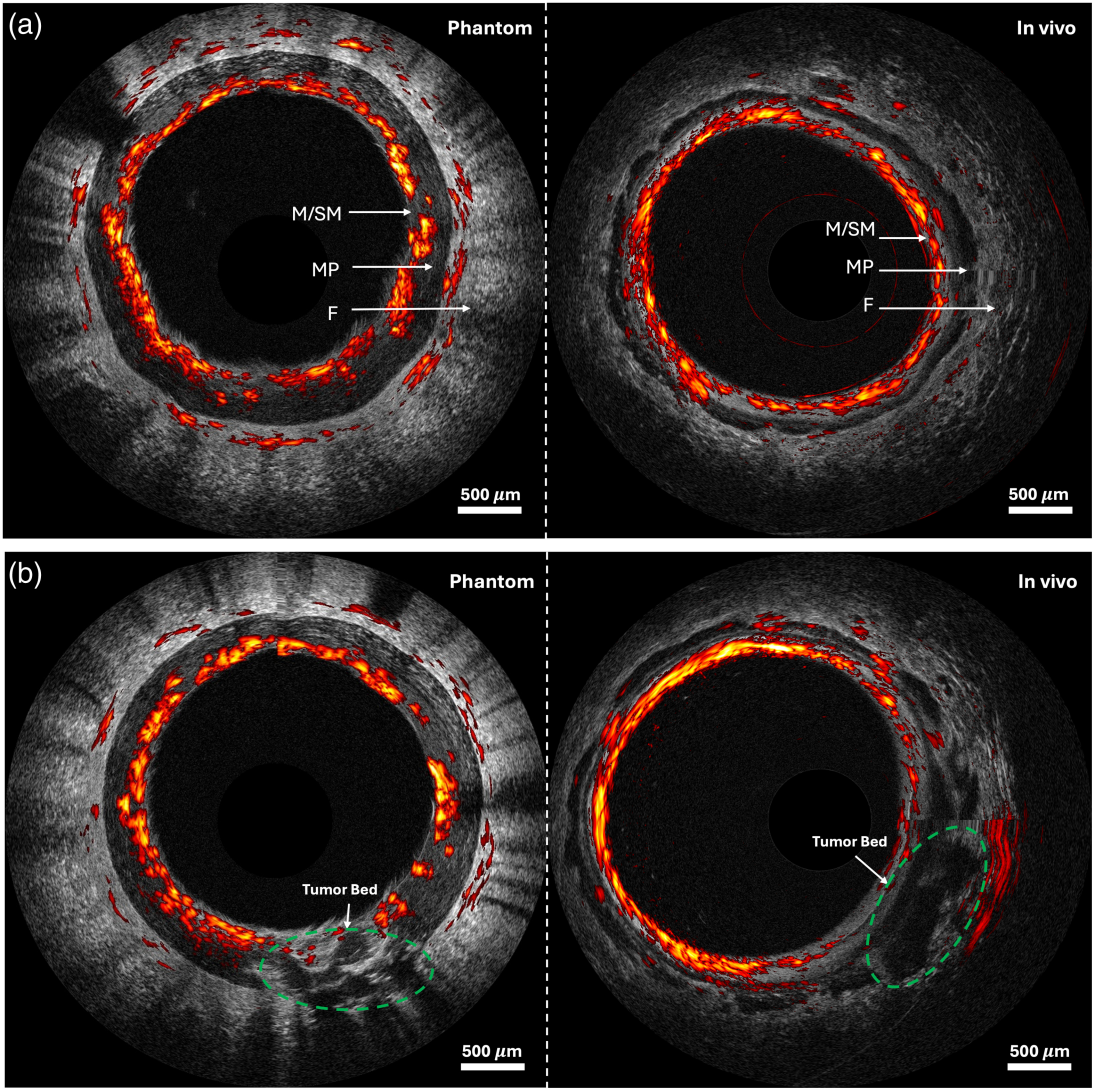
Examples of co-registered ARPAM/US images. (a) Normal region. (b) Cancer region. Scale bar: 500  μm. M, mucosa; SM, submucosa; MP, muscularis propria; F, perirectal fat. The *in vivo* patient image is from a 63-year-old male with invasive adenocarcinoma (1.1  cm×0.5  cm) treated with radiation and chemotherapy involving the muscularis propria. PA signals are primarily concentrated in the vascular-rich submucosa, with minimal signal observed in the muscularis propria and scattered vessels in the serosa. Reduced perfusion is evident in the cancerous region. In US images, normal tissue displays a continuous layered structure, whereas cancerous tissue exhibits invasion through the muscularis propria and disruption of normal layers. Additional patient images are published by Kou et al.^12^

The right column of Fig 7(a) illustrates the typical appearance of normal rectal tissue. Ultrasound (US) shows alternating hypo- and hyperechoic bands, corresponding from the innermost layer to the mucosa, submucosa, muscle, and fat of a normal human rectum. This distinct layered US feature was successfully reproduced in the loofah phantom. From *in vivo* acoustic-resolution photoacoustic microscopy (ARPAM) imaging, we observed uniformly high photoacoustic microscopy (PAM) signals originating from dense microvessels in the submucosa, and this is successfully mimicked in the first layer of the loofah phantom by the inked loofah vasculature. In addition, to mimic deep tissue vessels sometimes observable during *in vivo* imaging, thicker but sparser loofah vasculature was placed in the third layer of the loofah phantom and sporadic signals from these loofah fibers highly resembled what was observed in clinical studies.

In Fig. 7(b), a tumor phantom fabricated with a lower dose of chromophore is grafted onto the layered loofah phantom, mimicking a tumor that originates in the mucosa layer and invades through the muscularis propria into the perirectal fat. Compared with images of normal regions, both phantom and patient US images show disruption of the layered structure in tumor regions due to tumor invasion from the mucosa into deeper layers. Similarly, in ARPAM images of the patient, a sharp discontinuity in PAM signals was observed at the mucosa/submucosa interface, a pattern that was also replicated in the ARPAM images of the phantom. The representative co-registered ARPAM/US images of normal and cancer regions are obtained from a 63-year-old male patient.^12^

#### 3.2.2 3D reconstruction

**Fig. 8:**
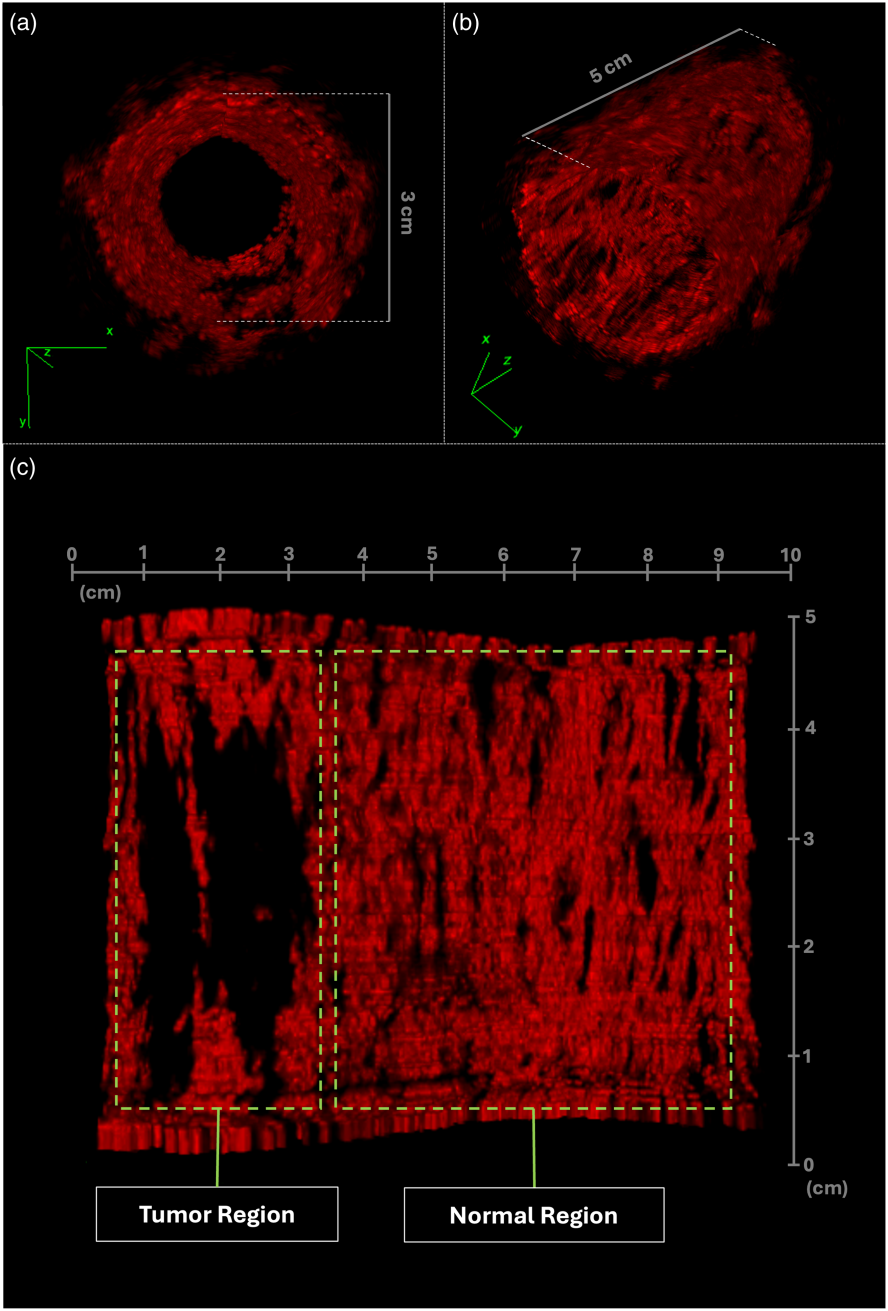
3D reconstruction of PA images. (a) Front views of the 3D reconstruction derived from polar images. (b) Side views of the 3D reconstruction derived from polar images. (c) Front view of the 3D reconstruction generated from unwrapped images.

The fiber distribution of the loofah vasculature is visualized by 3D reconstruction of consecutive ARPAM B-scans (Fig. 8). In the region designed to mimic normal colorectal tissue, uniform, and continuous distribution, PA signals were observed, matching the dense capillary network in the mucosal lining. Meanwhile, in the region designed to mimic a tumor after chemoradiotherapy, PA signals were indeed much attenuated. This observation was expected because the blood vessels are mostly destroyed after neoadjuvant chemotherapy. In addition, a sharp boundary between intended normal and tumor regions was observed, further demonstrating loofah’s capacity to emulate high-resolution complex tissue structures.

## 4 Discussion

In this study, we demonstrated, for the first time, loofah as a low-cost and practical material for making volumetric microvascular phantoms with submillimeter vascular details for photoacoustic imaging. Results from OR-PAM demonstrated loofah’s close resemblance to human vasculature on a microscopic scale. For potential clinical applications, we fabricated a tissue-mimetic loofah phantom to simulate endoscopic imaging in a clinical setting. We demonstrated that the endoscopic phantom produced images that closely resemble those collected *in vivo* from patients.

The natural variability of loofah fibers poses challenges for achieving consistency in phantom fabrication, as it is impossible to find two identical loofahs for perfect experimental reproducibility. However, as reported, loofahs of the same size and cultivar exhibit comparable natural variations. By selecting loofahs of similar size from the same cultivar, we can achieve relative consistency in experimental reproducibility. Furthermore, the subtle natural variability of different loofahs makes it ideal to mimic vascular distributions in complex biological tissues.

Despite the loofah’s elaborate submillimeter vascular network, it was challenging to mimic ultra-fine human capillary network with loofah. As shown in Fig. 5, the dominant diameter range is between 100 and 250  μm. In contrast, certain microvasculature, such as those found in the ovary and fallopian tube, often features regions with vessel diameters consistently under 50  μm.^13^ Among the loofah samples we purchased, we observed that some samples, particularly in the outer loop region, exhibited significantly smaller diameters, with a dominant range below 100  μm. This diameter variation may be attributed to differences in cultivars. Hopefully, future researchers with the necessary bandwidth and resources will study and analyze such diameter variations in cultivars. A common concern when building imaging phantoms from organic materials is their shelf life. Using a suitable embedding medium can also significantly extend the shelf life of the phantoms we create. Loofah itself, composed of stable xylem fibers, has an inherently long shelf life and can remain intact for months without degradation. However, embedding materials such as gelatin or agar require careful control of humidity and temperature to prevent deterioration during storage. Therefore, the shelf life of a loofah-based phantom is largely determined by the choice of the embedding medium, and selecting an appropriate material can greatly enhance the phantom’s durability and reusability. Based on our observations, loofah phantoms embedded in agar or gelatin were stored in sealed containers at 4°C in a refrigerator and exhibited no signs of degradation over the course of 1 month of experiments.

Here, we mainly demonstrated loofah’s structural resemblance to human vasculature and its ease of use as a phantom material. It is noted that loofah phantom can be extended to test the functional capabilities of photoacoustic imaging. Instead of using ink as the sole chromophore, employing a mixture of solutions with known spectral differences at various wavelengths allows for spectral unmixing and material decomposition. For example, ${\mathrm{NiSO}}_{4}$ and ${\mathrm{CuSO}}_{4}$ have been shown to serve as effective analogs for deoxyhemoglobin and oxyhemoglobin, respectively.^14^ However, both ${\mathrm{NiSO}}_{4}$ and ${\mathrm{CuSO}}_{4}$ are highly water-sensitive, and with gelatin and agar being hydrophilic, these sulfates can easily diffuse into the surrounding hydrogel. To tackle this challenge, there are ongoing efforts to replace agar with a suitable hydrophobic medium^15^ for embedding loofah. This would enable further exploration of using such fibers in quantitative photoacoustic imaging.^16^

## 5 Summary

In this study, we present the results of the construction and characterization of submillimeter microvascular loofah phantom for photoacoustic imaging. This loofah material as a carrier for a chromophore exhibits exceptional morphology and customizability to be shaped to vascular networks of different sizes and densities. Utilizing this material provides a new tool for photoacoustic system calibration and validation. In addition, data from loofah-based low-cost microvascular phantoms can provide needed realistic images to initially train machine learning models, thereby improving models’ robustness before further training and testing with clinical data.

## Data Availability

Data are available from the corresponding author upon reasonable request.
